# Data on composition and production rate of dental solid waste and associated management practices in Qaem Shahr, Iran 2016

**DOI:** 10.1016/j.dib.2018.05.114

**Published:** 2018-05-31

**Authors:** Monireh Majlesi, Nad Ali Alavi, Ali Akbar Mohammadi, Sima Valipor

**Affiliations:** a Department of Environmental Health Engineering, School of Health, Shahid Beheshti University of Medical Sciences, Tehran, Iran; b Department of Environmental Health, Neyshabour University of Medical Sciences, Neyshabour, Iran

**Keywords:** Dental waste, Biohazard waste, Domestic type waste, Chemical and pharmaceutical waste, Qaem shahr

## Abstract

This report investigates to analyse the production of waste in dental offices of Qaem Shahr city. In this study, from 120 dental offices in Qaem Shahr city, 21 offices were selected through random sampling. Sampling taken from 3 offices in 3 consecutive working days (on Sundays, Mondays and Tuesdays). The components were classified into three groups based on their specificity and potential. Total annual waste produced in Dentist Offices in Qaem Shahr city is 557.80 kg. In dental office, the amount of biohazard, chemical and pharmaceutical and domestic type wastes were 64.10, 2.70 and 33.20 respectively. Production percentages of biohazard, the highest weighted mean for potentially biohazard (155.25±0.63 g) and the lowest for chemical and pharmaceutical (6.35±1.85 g).

**Specifications Table**

**Table:** 

Subject area	Dental office in Qaem Shahr in Mazandaran Province
More specific subject area	Describe narrower subject area
Type of data	Tables and figure
How data was acquired	In this study, total amount of daily dental wastes from all parts of studied clinics were collected according to WHO guidelines, Basel convention provisions and Iranian national regulations
Data format	Raw, Analyzed
Experimental factors	The mentioned parameters above, in abstract section, were analyzed according to the production rates of different components of generated dental wastes and completed questionnaires. Sampling was carried out at 3 offices in 3 consecutive working days (on Sundays, Mondays and Tuesdays). Samples were manually separated and weighed to 46 parts. The components were classified into three groups based on their specificity and potential. The data were analyzed using Excel software and the share of each component and each category of three categories of waste was determined. A checklist containing 8 questions was used to evaluate the management of dental wastes.
Experimental features	The composition and production rate of dental solid waste and associated management practices were determined.
Data source location	Qaem Shahr, Mazandaran province, Iran.
Data accessibility	The data are available whit this article

**Value of data**

• Based on the data analysis, the maximum production rate is related to biohazard and potentially infectious wastes in dental clinics.
• Considering this research, particularly the biohazard wastes and their adverse effects on public health as well as the environment, proper management must be necessarily designed for efficient management and safe disposal of these wastes.
• No action was performed in order to reduce, segregate and recycle the dental wastes in these clinics.
• These data emphasize the demand for improving dental solid waste management, as well as waste reduction and recycling.

## 1 Data

The amount and percentage of production of various waste products in the whole dental offices of Qaem Shahr city are shown in Table 1. The total amount of waste produced annually in dental offices is 557.79 kg. The parts of the dental wastes consist of biohazard waste (64.09%), domestic type (33.20%), and chemical and pharmaceutical waste (2.70%). The rate of production of various components of biohazard wastes was presented in Table 2 and shows that the highest amount of weight and percent of saliva contaminated tissue napkins was 41.98 kg/year, which made up 74.7% of the waste and then it was a blood-contaminated cotton roll with weight 22.22 kg and 24.26% and in the third stage nylon gloves weighing 21.52 kg and 6.2%. According to Table 3, the total waste per head produced in dental offices in Qaem Shahr city is 242.22 g, with the highest mean weight for biohazard waste (155.25 g) and the lowest mean weight for chemical waste and pharmaceutical products (6.35 g). In Table 4, the most important results from the checklist used to study the management of waste dental offices in the city of Qaem shahr have been shown.

**Table 1:** Average of solid waste production in dental clinics in Qaem-Shahr city. Components of dental solid waste in dental clinics.

Type of waste	Yearly production (kg/year)	Percentage
Domestic-like wastes	185.2	33.2
Biohazard wastes	357.52	64.1
Chemical and pharmaceutical	15.08	2.7
Total	557.8	100

**Table 2:** Average of infection solid waste production in dental clinics in Qaem-Shahr city.

**Type**	**Amount (kg/year)**	**Amount**	**The cumulative**
			**percentage**
Saliva-contaminated tissues (infectious)	41.98	11.74	17.39
Saliva contaminated gauzes	19.15	5.36	27.22
Saliva-contaminated cotton	13.92	3.89	35.01
Blood-contaminated cotton rolls (infectious)	22.3	6.24	41.25
Saliva-contaminated cotton rolls (infectious)	14.76	4.13	45.38
Nylon gloves	21.52	6.02	51.4
Latex gloves	15.93	4.45	55.85
Suction tips	20.5	5.73	61.58
Needles, sharp objects and cutting blades (infectious)	16.01	4.48	66.06
Extracted teeth (infectious)	15.29	4.27	70.33
Dental mirror	15.32	4.29	74.62
Yarn Suture	5.22	1.46	76.08
Surgical blade	15.61	4.37	80.45
Suture needle	17.65	4.94	85.39
Wooden sticks (infectious)	16.1	4.5	89.89
Mouth thread	16.15	4.52	94.41
Serum chamber	19.98	5.59	100
Total (kg)	357.52	100

**Table 3:** Per capita waste for each patient in different parts of dentistry offices in Gaem-Shahr city.

Per capita waste per patient	Amount (g/day)
Biohazard wastes	155.25
Domestic-like wastes	80.6
Chemical and pharmaceutical	6.35
Total amount	242.2

**Table 4:** Management of solid wastes generated by dental offices.

Management method	Percent of clinics
Waste reduction programs	52.38
Solid waste separation programs	71.42
Waste recycling programs	66.66
Method of sterilization	71.4
Management of amalgam residues and chips	81
Management of sharp objects and cutting blades	100
Safety of sharp objects and cutting blades	0
Weighing and recording the amount of waste produced	33.3

## 2 Experimental design, materials and methods

### 2.1 Study area description

**Fig. 1:**
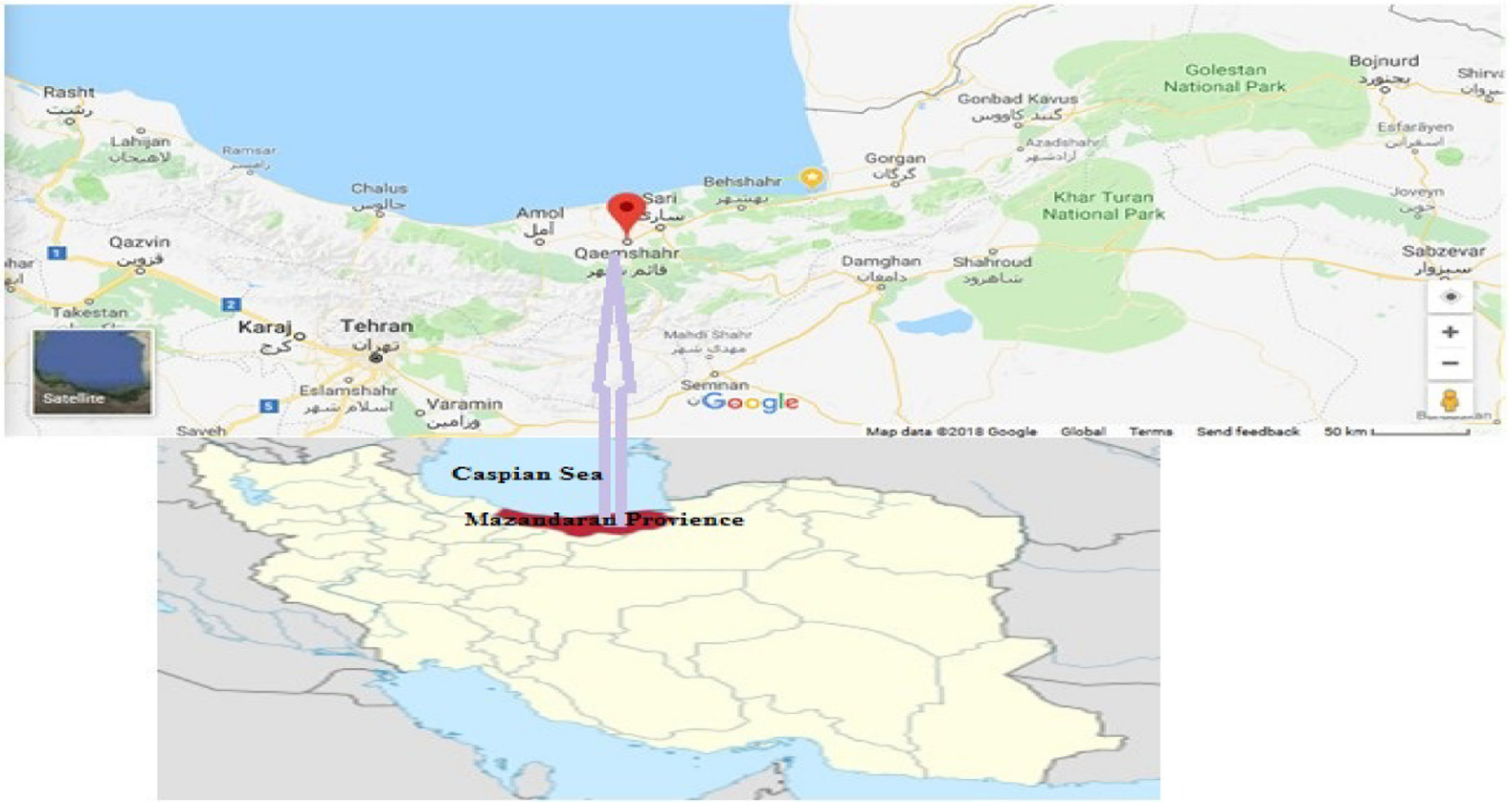
Map of study.

Qaem Shar is a county in Mazandaran Province in Iran. It is situated in 36°-28″ N and 52°-52′ ″ E with and area near 458.50 km^2^ Fig. 1.

In this cross-sectional study, 21 offices were randomly selected as sampling sites from the total of 120 dental offices in Qaem Shar since June to September 2016. Each sample is taken at the end of the day and three sampling steps were taken from each of 21 offices every three consecutive days (Sunday, Monday, Tuesday) each week so the total number of analyzed samples was 63. In this research, total amount of daily dental wastes from all parts of studied clinics were collected according to WHO guidelines, Basel convention provisions and Iranian national regulations, then, sorted with respect to constituents and placed into specified colored bags. In order to minimize the closure effect on waste production, weeks were chosen for sampling which there was no holiday during Saturday till Wednesday. Physical analysis and weighing were done at the end of each office hours and up to 10 h after sampling and transfer to a suitable place. At first, the waste sample was manually weighed into various components and weighed using the Mettler PM4000 (in milligrams) laboratory scale. Then weighted components were classified according to their specificity and risk aversion. Each section was weighed 3 times and finally the average of the numbers obtained for each section was considered. After weighing waste for the calculation of per capita waste, the amount obtained was divided by the number of people visited per day of work. In the next step, by averaging the numbers of three days of sampling from each office, the average daily production of the various components of the waste was determined in each office. In order to determine the annual amount of waste produced in dental centers, it was necessary that the average working days of the mentioned centers were held. It became clear that almost all units did not work on holiday days by asking dentists about this. Therefore, referring to the calendar, the average working days in year 2016 were 244. The amount of annual production of various waste components in 21 public dental offices were achieved by multiplying the average daily production of different components of waste in this number. Subsequently, it was decided to generalize the amounts received to the whole society (Qaem Shar city) by dividing the total number of dental units in the city (120 general offices), the number of sampling offices (21 general offices) was determined by the corresponding coefficient which was 71 for dental public clinics. The total annual waste production in all dental offices of Qaemshahr city was obtained by multiplying this coefficient in the amount of annual production of various waste components in general dental offices. In another part of the work, a checklist approved by the experts (containing 8 questions), the status of waste management in dentistry offices was investigated [1], [2], [3], [4], [5], [6], [7], [8]. Finally, the data were analyzed using SPSS 18 and EXCEL software.
