# Supplementary material for: Data on composition and production rate of dental solid waste and associated management practices in Qaem Shahr, Iran 2016
Source: Data Brief. 2018 May 31;19:1291–6. doi: 10.1016/j.dib.2018.05.114 (PMC6140284; doi:10.1016/j.dib.2018.05.114)
Supplement: Supplementary file 1 — Supplementary material [file mmc1.zip › Competing financial interests.pdf]

**Competing financial interests:**

The authors declare no competing financial interests.
